# Serological Detection of Rh-Del Phenotype among Rh-Negative Blood Donors at National Blood Center, Yangon, Myanmar

**DOI:** 10.1155/2020/3482124

**Published:** 2020-02-18

**Authors:** Saw Thu Wah, Saung Nay Chi, Kyi Kyi Kyaing, Aye Aye Khin, Thida Aung

**Affiliations:** ^1^Department of Medical Laboratory Technology, University of Medical Technology, Yangon 11011, Myanmar; ^2^National Blood Center, Yangon 11131, Myanmar

## Abstract

**Background:**

Red cell Rhesus (Rh) antigen expression is influenced by the genetic polymorphism of *RHD* and *RHCE* genes and reveals serologically different reactions of RhD variants such as partial D, weak D, and Rh-Del. Serologically, Rh-Del type can only be detected by an adsorption-elution technique, and it might be mistyped as Rh-negative. The prevalence of Rh-Del has not been reported yet in Myanmar.

**Method:**

A total of 222 Rh-negative blood donors in the National Blood Center were tested for weak D and Rh-Del by indirect antihuman globulin and adsorption-elution method, respectively. RhCE typing was performed among Rh-negative and Rh-Del.

**Results:**

Of them, 75.2% (167/222) were Rh-negative, 15.8% (35/222) were Rh-Del, and 9% (20/222) were weak D. Of 202 blood donors (167 true Rh-negative and 35 Rh-Del), all of the Rh-Del positives were C-antigen-positive with 94.3% Ccee phenotype (33/35) and 5.7% CCee (2/35). Most of the Rh-negative donors (80.2%) were ccee phenotype (134/167).

**Conclusion:**

About half of Rh-Del subjects were repeated donors, and attention was needed to avoid transfusion of truly Rh-negative patients to prevent alloimmunization. It is recommended to do Rh-Del typing of Rh-negative donors who are C-antigen-positive and consider moving them to the Rh-positive pool. Further study is needed to clarify the alloimmunization status for transfusion of Rh-Del blood to Rh-negative recipients. Molecular markers for RhD-negative and D variants should be established in the Myanmar population to improve selection of antisera for Rh typing and enhance safety of the transfusion services.

## 1. Introduction

The Rhesus (Rh) blood grouping system has five common antigens (D, C, E, c, and e), among which D antigen is the most immunogenic. Routine Rh blood grouping detects D antigen and phenotypes blood as Rh-positive or Rh-negative. D antigen is encoded by the *RHD* gene, while C, c, E, and e antigens are encoded by the *RHCE* gene. These genetic polymorphisms influence Rh antigen expression on the red cell surface that determine serologically different reactions of RhD variants such as partial D, weak D, and Rh-Del [1]. In the Rh-Del phenotype, D expression is so low that routine serological testing mistypes it as Rh-negative. The phenotype can only be detected currently by adsorption-elution technique [2]. In Asian populations, up to 30% of Rh-negative blood units by routine serological testing are found to be Rh-Del [3]. To date, the frequency of Rh-Del among Rh-negative blood donors has not been reported yet in Myanmar.

If Rh-negative individuals receive Rh-positive blood, up to 80% of them produce alloantibody D [4]. Although the Rh-Del phenotype expresses a very low density of D antigen on red cells, several reports indicate that it can cause both primary and secondary alloimmunization in Rh-negative individuals after transfusion. The evidence of primary alloimmunization was reported in Chinese and Korean and secondary alloimmunization in Chinese and Japanese people after Rh-Del transfusion to Rh-negative patients [5–8]. Thus, it is crucial to detect the Rh-Del phenotype among blood donors reported to be Rh-negative in order to prevent alloimmunization and subsequent hemolytic transfusion reactions.

A descriptive observation study was conducted in blood donors who were phenotyped as Rh-negative by routine serological tests. The objective of this study was to determine the frequency of Rh-Del phenotype among the Rh-negative blood donors. Furthermore, it has been found that the Rh-Del phenotype is closely related to RhC+ and some RhE + variants [9, 10]. In order to assess the possible association between the Rh-Del phenotype and other Rh antigens, Rh C, c, E, and e, phenotyping was performed by serological testing. In doing so, this study expected to recognize the Rh-Del phenotype among RhC+ and/or RhE + individuals. It was hoped that this study would provide valuable information for better management of transfusions among Rh-negative blood recipients.

## 2. Methods

### 2.1. Study Design and Area

A cross-sectional laboratory-based descriptive study was conducted in 222 Rh-negative voluntary blood donors at the National Blood Center, Yangon, Myanmar. The history of blood donation in all donors was collected. This study was approved by the Institutional Review Board of the University of Medical Technology, Yangon (IRB approval no. 1/2018-1). One ml of left-over blood, collected in EDTA pilot bottles, was taken from known Rh-negative blood donors serotyped by the immediate spin tube agglutination test.

### 2.2. Indirect Antihuman Globulin Test for Weak D

One drop of 3–5% washed red cells in normal ionic strength saline was mixed with two drops of monoclonal blend anti-D (IgM + IgG) (Novaclone, Dominion Biologicals Ltd, Canada) into a tube and incubated at 37°C for 1 hours. After incubation, agglutination was read. Then, the red cells were washed 3 times with normal ionic strength saline, and the tube was drained upside down on the tissue paper. Two drops of antihuman globulin (AHG) (Biotech, UK) serum was added and spin at 3000 rpm for 15 secs. The agglutination was read microscopically, and positive agglutination was recorded as weak D. In order to exclude false positive due to in vivo sensitization of red cells with incomplete antibody, direct antihuman globulin test was performed in all weak D sample. Samples showing negative antihuman globulin test were then underwent adsorption-elution test for Rh-Del detection.

### 2.3. Adsorption-Elution Technique for Rh-Del

Equal volumes (200 *μ*L) of washed red cells and monoclonal blend anti-D (IgM + IgG) (Novaclone, Dominion Biologicals Ltd, Canada) were mixed and incubated at 37°C for 1 hour. Then, red cells were washed thoroughly with normal ionic strength saline, and the last washed supernatant was kept for testing. The elution reagent was prepared in-house (acid-glycine/EDTA (0.1 M glycine-HCL buffer (pH 1.5); 10% EDTA with ratio of 4 : 1). Equal volumes (200 *μ*L) of packed, washed sensitized red cells and elution reagent were incubated at room temperature (22–24°C) for 1 minute (over incubation was avoided to prevent damage to the RBCs). Then, 28 *μ*L of 1 M TRIS-NaCL was added, mixed, immediately centrifuged at 1000 rpm for 1 minute, prior to collection of supernatants (eluate). The eluate pH was adjusted by 1 M TRIS-NaCL; the optimal pH should be between 7.0 and 7.4. The eluate and last washed supernatants were used for indirect antihuman globulin testing against Rh-positive and Rh-negative control cells. Eluate that showed agglutination with Rh-positive cells was interpreted as Rh-Del, and no agglutination as Rh-negative. Supernatants acted as negative controls and should show no agglutination with both Rh-positive and Rh-negative cells. Coomb's control cells were added to all negative agglutination results to exclude false negative [11].

### 2.4. RhCE Typing

The monoclonal antibody of anti-C and anti-e (fortress diagnostic Limited, United Kingdom) and anti-c and anti-E (National Blood Center, The Thai Red Cross Society, Thailand) were used for RhCE typing by immediate spin tube method.

### 2.5. Statistical Analysis

Data were analyzed by using Statistical Package for the Social Sciences (SPSS) version 16.0. *p* value less than 0.05 was determined as statistically significant.

## 3. Results

A total of 222 Rh-negative voluntary blood donors from the National Blood Center, Yangon, were recruited in this study. RhD antigen status was determined by serological techniques (immediate spin agglutination technique, indirect antihuman globulin technique and adsorption-elution technique), and most of the Rh-negative blood donors were confirmed as Rh-negative (167/222; 75.2%). However, 15.8% (35/222) of the Rh-negative blood donors were detected as Rh-Del phenotype and 9% (20/222) as weak D phenotype. In terms of ABO grouping, blood group O was the most common ABO blood group (81/222; 36.5%) followed by blood group B (74/222; 33.3%), group A (56/222; 25.2%), and group AB (11/222; 5%). Male donors were predominant and made up 62.2% (138/222) of the study population.

In order to determine the relationships between the Rh-Del phenotype and other Rh antigens, phenotyping for Rh C, c, E, and e antigens were performed among 202 blood donors (167 truly Rh-negative and 35 with Rh-Del phenotype). Of these donors, 67.8% (137/202) were phenotype ccee, 27.2% (55/202) Ccee, and 5% (10/202) CCee. No E antigen was detected among Rh-negative or Rh-Del subjects. All Rh-negative and Rh-Del donors were antigen E-negative and antigen e-positive. Unfortunately, RhCE typing was only performed in six of the twenty weak D donors. Among them, three were Ccee, while the other three were CCee, CCEe, and CcEe (one each).

Table 1 shows the distribution of RhCEce antigens among the 202 Rh-negative and Rh-Del donors. All Rh-Del donors (35/35) were antigen C-positive, while Rh-negative were mostly antigen C negative (134/167; 80.2%), a statistically significant difference (*p* < 0.0001). Regarding antigen c type, there was no significant difference between the two RhD phenotypes (*p*=0.685).  Table 2 presents the association of RhD phenotypes (Rh-negative and Rh-Del) with RhCEce phenotypes. RhD-negative donors were mostly ccee phenotype (134/167; 80.2%), and Rh-Del were mostly Ccee phenotype (33/35; 94.3%), followed by CCee (2/35; 5.7%), differences which were significantly different (*p* < 0.0001).

Table 3 shows the distribution of Rh D phenotypes among ABO blood groups. There was no significant association between the two blood group systems (*p*=0.444).  Table 4 presents the frequency of gender and number of blood donations in Rh-negative and Rh-Del donors. Nearly half of the Rh-Del group (17 out of 35) was repeat donors.

## 4. Discussion

Serological detection of Rh-Del and weak D phenotypes was performed among 222 Rh-negative voluntary blood donors from the National Blood Center, Yangon. The prevalence of Rh-Del was 15.8% and weak D 9% among the study population. This finding was consistent with that of other Asian countries, as documented in Korean (17.9%), Thai (20%), and Chinese (20.6%) populations [12–14]. However, Rh-Del was found in only 2.3% and 1.5% of Malaysian and north Indian populations, respectively [15, 16]. Similarly, Rh-Del was found in 0.1% of Germans and less than 1% of Austrians and Brazilians [17, 18]. This difference in prevalence is thought to be due to the diversity of ethnicities.

In this study, there was a limitation in serotyping of RhCEce antigen among all of the weak D donors. Since weak D typing by the indirect antihuman globulin method is done in most laboratories for confirmation of Rh D status, detection of weak D phenotype might not be a problem in transfusion medicine. However, Rh-Del phenotype status is not routinely assessed, and there might be misidentification of those donors as Rh-negative. There have been several reports of primary and secondary alloimmunization in Rh-negative individuals after transfusion or pregnancy with Rh-Del, even though the density of D antigen on red cells is very low [5–7]. So, it is important to pay attention to this matter in transfusion practice in order to prevent alloimmunization.

Regarding the RhCE phenotypes among those who are Rh-negative and Rh-Del, ccee was the most common phenotype in this study followed by Ccee and CCee. This finding was consistent with that reported in Thai and Chinese populations [13, 14]. It is reported that Rh-Del phenotype is associated with Rh C or E positivity [9]. In order to determine the relationship between Rh-Del and RhCE antigens, antigen typing was performed by serological techniques and found that all Rh-Del donors were C-antigen-positive (100%). This was statistically different (*p* < 0.0001) from that in donors with the Rh-negative phenotype who were mostly c-antigen-positive (80.2%). In those with the Rh-Del phenotype, Ccee was the most common phenotype (33/35; 94.3%), followed by CCee (2/35; 5.7%). This finding was similar to that reported in a Chinese population [14]. Interestingly, the ccee phenotype was found in Rh-Del individuals in German and Austrian populations [17, 18]. E antigen was only positive in donors with the weak D phenotype in our study population.

Since Rh and ABO antigens are derived from different chromosomes, it was not surprising of no significant association between the two blood group systems (*p*=0.444) were observed. Nearly half of the Rh-Del group were repeat donors, so there was a chance of causing alloimmunization in patients truly Rh-negative. Such blood should be considered as Rh-positive to prevent this.

## 5. Conclusion

To sum up, 15.8% of apparently Rh-negative donors were found to be Rh-Del in our study population. All of Rh-Del positive donors were C-antigen-positive. For safe blood transfusion, Rh-Del typing should be done among all Rh-negative donors who are RhC-positive. In order to prevent RhD alloimmunization in blood recipients, this study recommend that Rh-Del phenotype donors be managed as part of the RhD-positive pool. Further study is needed to determine the alloimmunization status of Rh-negative patients who have received Rh-Del transfusions. Moreover, molecular detection of Rh-Del variant has been developed by the researchers, and they found that *RHD* K409K is the most common variant in Asian population. As molecular screening for Rh-Del variant has become routine service in Central Europe, molecular testing for detection of *RHD* K409K together with RhC typing should be considered mandatary among Rh-negative donors in Asian countries [2]. Since serological techniques are tedious, time consuming, and have a high possibility of false-negative or false-positive results, it is important to develop genotyping technique as a routine practice. An alternative practical approach could be to perform Rh-Del typing by solid phase method [19]. In a routine service, it is essential to elucidate the most commonly encountered alleles for Rh-negative, Rh-Del, and weak D phenotypes in each population like ours. In doing so, *RHD* detection can be done confidently among Rh-negative donors by multiplexed molecular technique to enhance transfusion safety.

## Figures and Tables

**Table 1:** Distribution of RhCEce antigens in Rh-negative and Rh-Del phenotype (*N* = 202).

RhCEce antigens		Rh-negative	Rh-Del	*p* value
		*n* = 167	*n* = 35	
C	Positive	33 (19.8%)	35 (100%)	<0.0001^1^
	Negative	134 (80.2%)	0 (0%)	

E	Positive	0	0	
	Negative	167 (100%)	35 (100%)	

c	Positive	159 (95.2%)	33 (94.3%)	0.685^2^
	Negative	8 (4.8%)	2 (5.7%)	

e	Positive	167 (100%)	35 (100%)	
	Negative	0	0	

^1^Chi-squared test, ^2^Fisher's exact test.

**Table 2:** Association of RhD phenotypes among RhCEce phenotype (*N* = 202).

RhCEce phenotype	Rh-negative	Rh-Del	*p* value
	*n* = 167	*n* = 35	
ccee	134 (80.2%)	0 (0%)	<0.0001^1^
Ccee	25 (15%)	33 (94.3%)	
CCee	8 (4.8%)	2 (5.7%)	

^1^Chi-squared test.

**Table 3:** Association of Rh-negative and Rh-Del among ABO blood groups (*N* = 202).

		Rh-negative	Rh-Del	*p* value
		*n* = 167	*n* = 35	
ABO blood groups	A	44 (26.3%)	10 (28.9%)	0.444^1^
	B	52 (31.1%)	13 (37.1%)	
	AB	7 (4.2%)	3 (8.6%)	
	O	64 (38.3%)	9 (25.7%)	

^1^Chi-squared test.

**Table 4:** Distribution of gender and blood donation number in Rh-negative and Rh-Del donors (*N* = 202).

		Rh-negative	Rh-Del
		*n* = 167	*n* = 35
Gender	Male	103 (61.7%)	26 (74.3%)
	Female	64 (38.3%)	9 (25.7%)

History of blood donation (number of blood donation)	1	77 (46.1%)	18 (51.4%)
	2	36 (21.6%)	4 (11.4%)
	3	13 (7.8%)	5 (14.3%)
	4	12 (7.2%)	3 (8.6%)
	5	6 (3.6%)	1 (2.9%)
	6	5 (3.0%)	1 (2.9%)
	7	4 (2.4%)	0
	8	4 (2.4%)	0
	9	1 (0.6%)	2 (5.7%)
	10	5 (3.0%)	1 (2.9%)
	11–13	4 (2.4%)	0

## Data Availability

The data used to support the findings of this study are available from the corresponding author upon request.
